# International perspectives on gaps and solutions for integrating research evidence into classroom practices

**DOI:** 10.1038/s41539-025-00370-x

**Published:** 2025-11-19

**Authors:** A. E. J. Bowen, R. A. Ferreira, A. Tolmie, M.S.C Thomas, G. Borst, J. Van Herwegen

**Affiliations:** 1 Centre for Educational Neuroscience, University of London, London, UK; 2 School of Psychological Sciences, Birkbeck College, University of London, London, UK; 3 Millenium Nucleus for the Science of Learning, Talca, Chile; 4 Department of Psychology and Human Development, Institute of Education, UCL’s Faculty of Education and Society, London, UK; 5 LaPsyDÉ, Université Paris Cité, CNRS, Paris, France

**Keywords:** Psychology, Social sciences

## Abstract

A persistent difficulty in the field of science of learning is translation of findings into the classroom. Five heads of research labs involved in the science of learning from three different countries (the United Kingdom, France, and Chile) conducted a workshop to discuss challenges for translation in the science of learning field. Presentation slides and notes were thematically analysed using an adapted framework by Miles (2017) to produce a gap analysis. Gaps were identified in relation to: (1) existing theory and knowledge; (2) the research-practice divide; (3) research methodologies; (4) empirical testing and evidence verification; and (5) research with specific populations. Examples from work across the labs illustrated international perspectives on challenges and evidenced possible solutions. Strategies for progress included research into lesser-studied areas and populations, replicating or repeating intervention evaluations, and establishing research partnerships between educators and research institutions.

## Introduction

The purpose of integrating educational methods informed by research evidence into classroom settings is to improve students’ learning outcomes by implementing the most effective strategies to support learning. The *science of learning* is a relatively new field of research broadly concerned with this area; while diverse definitions of the field and its aims have been proposed^1^, this article’s working definition is that research in the science of learning aims to integrate knowledge of the processes by which learning occurs in the brain with cognitive and educational theories to provide a comprehensive explanation for learning. To address this aim, it brings together transdisciplinary expertise from researchers and practitioners across the fields of neuroscience, psychology, and education in order to provide teachers with usable knowledge on how to improve learning outcomes^2^.

In general, awareness of the reasons and need for evidence-informed practices in education is higher than ever, and educators demonstrate willingness and interest to engage with research evidence^3–5^. Previous research findings suggest that research-informed practices play an important role in fostering improved student outcomes, but consistent improvement is often dependent upon levels of resources, buy-in from stakeholders, and the implementation qualities of practices^6,7^. Large-scale synthesis of research findings has indicated which practices generally work better than others to support outcomes^8,9^. Some individual studies have also highlighted practices which are ineffective or potentially harmful for certain profiles of students – important limitations which may otherwise be lost in the enthusiastic uptake of new practices, such as in the case of universal mindfulness interventions^10^. Despite the value that research can offer for practice, classroom practices and teacher training remain largely uninformed by research^11,12^, though in the United Kingdom, there has been some recent movement and momentum towards integrating more research evidence into Initial Teacher Training and Early Career Framework syllabi^13^. It is therefore evident that while some momentum appears to be building for evidence-informed practices in education, challenges remain for evidence integration, and these must be addressed to support the uptake of research-informed classroom practices.

The ‘research-practice gap’ refers to the difficulties of integrating theory, based on research, into practice in an applied setting^14^. There are many factors in the research-practice gap, some of which relate to differences between scientists and practitioners in terms of understanding each other’s priorities and lack of a shared language or knowledge base^15^. For example, in educational contexts, researchers are typically concerned with whether a strategy ‘worked’ in general, usually based on evidence from rigorous experimental investigation across different settings and children, whereas educational practitioners are more interested in the question of ‘Will it work in my specific classroom or context?’, taking the theoretical implications of research and applying it in the context of their specific educational practice and learner needs^3,16^. This presents a fundamental difference in the meaning of research for those conducting it and those it is intended for, which carries implications for rethinking how research is conducted from the ground up if its intention is to meet the needs of practitioners. In addition, from the educator’s perspective, engaging with research is a luxury not often afforded due to the more pressing concerns of workload and managing students in the midst of a restrictive funding landscape^17^. Integrating research is therefore about far more than willingness to engage in research-based or informed practice: there are multi-level influences on educators’ engagement with research, including institutional, policy and system-related factors^18,19^. Personal motivation is just one part of a much broader picture.

Gap analysis is a method that can be used to identify some of the factors regarding the disconnect between research and practice, as well as potential methods to address them^20^. While researchers are typically trained and encouraged to spot unexplored topics in existing fields, the process of identifying gaps is typically unstandardised and unsystematic. However, some researchers have devised methods for systematically conducting research gap analysis^21,22^. A gap analysis framework proposed by Miles^23^ presents a theoretical model which adapts and expands on previous models. The framework proposed by Miles defined and conceptualised seven different types of research gaps for the purpose of developing agendas for research which can address these different types of gaps. Based on a review of literature for this study, it does not appear that this framework has been applied to define an agenda for research in the field of education to date. While the research-practice gap is just one dimension of this framework, other dimensions, such as gaps in theory and empirical testing, can contribute to issues with integrating evidence in practice, therefore it is important to examine a range of gaps and identify what their relevance is for future work to support evidence integration in education.

As an additional layer, while issues relating to the research-practice gap may be common across multiple settings and fields (e.g. the health field^14^), their context in education has not been directly examined from an international perspective. The roles of cultural factors, contextual factors, policy priorities, and the presence or absence of supporting systems (for example, data sharing, communication, and existing partnerships between schools and research institutions) are all potential influencing factors in effective research integration. Exploring these factors across different nations permits identification of gaps through exploring the similarities and differences between contexts, as well as best practices to address issues around research-informed practices that may cut across different contexts.

This paper presents consensus in discussion between researchers from institutions in the United Kingdom, France, and Chile on current gaps concerning the integration of research evidence into educational practice. Using an adapted version of Miles^23^ as an organising framework, current gaps and possible solutions were identified, with examples of contextual variations by country given where relevant.

## Results

### Gaps in existing theory and knowledge

As identified in workshop discussions and supported by existing literature, the theoretical basis for classroom practice has remained roughly the same for decades, with teacher training drawing largely upon Piagetian^24^ and Vygotskyian^25^ cognitive and social learning theories and little else^26,27^. Perhaps due to a lack of viable, evidence-informed alternatives, some debunked theories also persist in teacher training; one notable example of this is continued use of *learning styles* models (particularly Visual-Auditory-Kinaesthetic, or VAK), which an overwhelming majority of teachers continue to report believing in ref. ^28^ despite robust evidence that using practices based on learning styles theory does not have a positive impact educational outcomes, and may have a detrimental impact^29,30^.

In terms of the evidence base for education, the distribution of research across different research areas remains sharply uneven. Traditionally, there has been a strong research focus on language-related issues or research about learning to read^31^, some focus on maths-related issues (increasing in recent years, but still much less than reading), a small amount on science, and a negligible amount on other areas of the curriculum, as supported by evidence from bibliometric and review studies^32,33^. In addition to this, there has been a much greater focus on early years and primary education over secondary education, and research on adult learning has been more or less entirely absent^33–35^. Also, for specific student populations there is an imbalance of knowledge or research evidence. Whilst there is some research evidence on how reading and mathematical abilities can be supported in students with dyslexia or those with mathematical learning difficulties, evidence around what works for students Special Educational Needs and Disabilities (SEND) in other areas of education is scant^36^.

This unevenness is driven to some extent by priority, given the importance of early education for future outcomes^37^ and the importance of reading for accessing the wider educational curriculum. However, it is also driven by biases in funding organisations that mainly focus on funding basic research—which are partly the result of lack of knowledge within those organisations^38,39^, but also by political drivers. In England, reading, writing and mathematics are the only formally assessed areas of the curriculum in primary school, hence the overriding concern with performance and assessment of these subjects. Unevenness in the research landscape is also promoted by lack of coordination in priority-setting, though this is gradually changing; for example, the research agenda of autism funding body Autistica is totally informed by its stakeholders at all levels^40^.

The contrasting situations of educational research in France and the United Kingdom provides an example for the mediating role of system factors in the theory gap. In the United Kingdom, organisations such as the Education Endowment Foundation (EEF) and the What Works Clearinghouse (WWC) have existed for more than a decade now with the purpose of promoting evidence integration into education and the development of standards for developing and testing theories and interventions, usually prioritising Randomised Controlled Trial (RCT) or other experimental models. No such organisations exist in France, and perhaps as a result, there has been very little large-scale experimental work and knowledge across all aspects of the curricula is lacking, resulting in a paucity of evidence that could be used to inform policy makers in France.

In Chile, organisations dedicated exclusively to integrating research evidence into education, particularly through large-scale experimental methods such as Randomised Controlled Trials (RCTs), are notably absent. However, several foundations focus on advancing education and promoting teaching practices informed by scientific evidence. For example, Fundación Arrebol (https://fundacionarrebol.cl/) supports education grounded in scientific research, while Mentes Transformadoras (https://www.loligo.cl/mt) aims to bridge the gap between science and teaching. The primary role of these foundations is the dissemination of knowledge and the provision of lifelong teacher training, rather than producing rigorous research such as RCTs.

Notably, recent efforts have aimed to address this gap. Nodo CTCI, through the Consorcio de Investigación y Evidencia Educativa (https://www.ctci.cl/consorcio-educacion/), has been established with the objective of generating research that is directly relevant to education. This consortium serves as a bridge between universities and research centres, policymakers, and practitioners, facilitating collaboration to ensure that research aligns with the practical needs of the educational sector. While still in its early stages, this initiative represents an important step toward creating an evidence base to inform educational practices and policies in Chile.

### Gaps between research and practice

In education, the distance between theory and practice is at once as fine as a hair and as wide as a gorge. Teachers consistently express desire to work in a theory- and evidence-informed way^5,17,41^, as they recognise the importance of providing the best support available for their students. Ultimately, integration of research into classroom practices should make their work easier and more successful, and any teachers believe that they are already working in evidence-informed ways^3,42–44^. However, due to previously discussed issues with the knowledge base, not all practices have been evaluated by rigorous research. Lack of knowledge, for example in relation to special educational needs, may in turn confer a lack of confidence working with these populations of students^3^. In addition, some teachers may be aware of the limits of their current knowledge or practice but feel frustrated and inhibited by policies coming from school level and above, which in the UK are usually set to meet the agenda of regulatory bodies such as Ofsted^34,45^. The consequences of failing to meet Ofsted requirements confers an aversion to risk-taking, instilling a mindset in teachers and schools of status quo maintenance rather than a mindset of improvement and generativity^34^.

Failure to properly mobilise or translate some forms of knowledge into the classroom is one reason for the gap between research and practice. For example, the neuroconstructivist approach to learning provides an alternative positioning to purely cognitive or social explanations for how and why learning happens, emphasising the role of developmental processes in the relationship between brain and cognitive development in children. Under this theoretical approach, learning can be seen as a reflection of progressive increases in the complexity of cortical structures and the sophistication of associated cognitive representations^46–48^. Whilst some cognitive theories, such as working memory and the concept of developing increasingly complex representational models, have entered the recent Initial Teacher Training Core Content Framework in the UK^13^, the extra mile of developmental, integrative, neuroscience-informed (i.e. neuroconstructivist) explanations for learning have not yet penetrated teacher education. This may be partly because the theory itself is complex, but also that the principles do not appear immediately practical or actionable. Teachers can easily understand and relate to theories such as scaffolding (Vygotsky), because they can see for themselves how learners advance based on prior knowledge and require materials appropriate to their developmental stage to advance (Piaget). Though it is easy enough to make the connection that activity in the brain, as the organ of learning, relates to how students learn, it is not immediately possible for teachers to monitor the brain processes or to directly and reliably see how learning affects changes in brain activity. In the absence of a cohesive strategy for translation or application, theories which attempt to connect different levels of explanation for learning and include a neuroscience-informed perspective therefore remain distant from day-to-day life in the classroom.

In addition, existing theories that have made some headway to classroom translation have a relatively narrow focus: working memory focuses on memory processes, cognitive load on capacity, self-regulated learning on metacognitive processes. There is no theoretical integration (or only very limited integration) between these. This has a knock-on effect on classroom approaches, as teachers are left without a full, dynamic and interconnected picture of how cognitive development and learning happens, and how it can be different in neurodiverse learners. When teachers and school leaders aren’t provided with a more complete picture of understanding, this may in turn have a knock-on effect on the types of evidence-informed initiatives being introduced, as interventions tend to prioritise small piecemeal changes within specific subjects, and often for specific profiles of students, rather than developing overall interconnected pedagogical strategies to support learning outcomes for all students. Such strategies should be informed by pedagogical practice as well as learning theories, necessitating collaboration and dialogue between practitioners and theorists.

One of the greatest barriers to evidence informed practice is that teachers usually do not have access to literature or training in research literacy, preventing them from researching independently to develop their own practice^49–52^. Educators also require sufficient bandwidth to engage with research, and potentially, an element of incentivisation that goes beyond the nebulous notion of ‘improved outcomes’. Whilst in the United Kingdom Ofsted used to incentivise research engagement, this has not been the case for some time now, meaning that schools which form research partnerships (such as in those in the EEF’s research schools network) are generally those which are already high performing and have the extra resources required to engage^53^. A recent poll from the United Kingdom^17^ reported that workloads were teachers’ greatest factor of concern, followed by funding cuts and student behaviour. In the same report, teachers likewise reported that student behaviour was the greatest challenge to supporting student learning. If it were possible to address these issues — lighten the workload, reduce some of the students’ behavioural challenges, reduce systemic stress from funding concerns, and reintroduce incentivisation for schools to engage with research — the priority and capacity for research integration would increase. These are not easy asks by any means, but the longer these contextual issues remain unacknowledged and unaddressed, the greater the gulf for educational researchers and practitioners to overcome. However, this is not to suggest that we must wait for the right conditions to be present before implementing evidence-informed practices in schools; indeed, implementing evidence-informed practices could play an important and valuable role in addressing some of these contextual issues as they currently stand, such as in the provision of strategies to address the challenges of behavioural management and streamline practices to reduce workload for teachers.

Within our team, there were examples of successful outreach efforts used to engage educators with research. For example, prominent Chilean neuroscientists, such as Marcela Peña, Pedro Maldonado, and Francisco Aboitiz, have participated in public awareness events, including seminars and talks aimed at the general public and educational community^54–56^. These efforts are complemented by master’s programmes and diplomas in educational neuroscience or the science of learning offered by several Chilean universities^57–59^. More recently, in 2024, Roberto Ferreira was invited to present the work of the Millennium Nucleus for the Science of Learning on *Exploradores, del átomo al cosmos*, a science television programme broadcasted by National Chilean TV. This episode is available online for viewing^60^.

Similar efforts are being pursued in London, where the CEN hosts weekly seminars for an audience of researchers and practitioners; these seminars are hosted at 16:00 when teachers are able to attend after the end of school time. The live CEN seminars typically attract an audience of 50–100 per session and recordings are uploaded to YouTube to reach an even wider audience. A Master’s degree in educational neuroscience is available as a joint venture between Birkbeck College, University of London, and the UCL Institute of Education. Intake on this course is around 20 students per year and the course largely attracts teachers, teaching assistants and school leaders. Educational neuroscience modules are also becoming increasingly available on courses at other universities. As essential as these efforts by individual researchers and institutions are, it is difficult to promote the widespread change necessary with only small pockets of dedicated efforts rather than a coordinated strategy between multiple organisations.

### Gaps in research methodologies

The role of research translation, outreach, and developing a shared language between stakeholders may be moot, if the research being conducted is fundamentally not meeting the needs of educators. Moreover, even the most well-developed intervention may fail, if it cannot adapt to the contextual demands of classrooms at scale. In terms of methodology, these facts necessitate a rethinking of the way research is designed and conducted from the ground up, demanding research that is built around the needs of schools and classrooms with educators’ priorities in mind.

There are multiple implications here. Perhaps most salient is that the epistemological approach of educational research needs to be adjusted to include the knowledge of teachers and educators. Participatory methods can be used to discover their concerns, shape these into research questions or testable hypotheses, and receive feedback on what is/is not possible in their contexts in terms of study and intervention design^61^. Even at the stage of efficacy testing, the role of contextual factors in intervention processes and mechanisms should be taken into account so that an intervention can make the transition to effectiveness trial at scale^62^ — and there is no better way of discovering contextual factors than by engaging with teachers. In addition, strategies have been proposed to better conduct research using practice-based insights and for teachers to be trained to conduct their own rigorous research^63^, but examples of research grounded in practice or conducted by educational practitioners remain rare^64^.

In terms of shaping research methods to facilitate translation, it must be recognised that the questions that educators want addressed may not be the questions that research into theory or interventions can answer. This has facilitated the perception amongst some teachers that research may have limited relevance to their classroom practice^3,65–67^. Teachers typically need theoretical principles which have a level of granularity that allows them to be applied to specific situations, specific questions, specific populations of students. Research questions are typically too broad and general to answer these questions. The CEN gathered data on this topic as part of a study which interviewed CEOs in educational intervention provision^34^, and this issue is highlighted in a revealing quote by one interviewed CEO from this sample:

*I think a lot of the academic research tends to be quite broad, quite large scale, and a lot of aggregated findings, whereas actually what we’re trying to do with teachers is change their in-the-moment perception, action, reflection on much smaller, much more granular things. In general, you don’t get academics spending a lot of time researching “Which of these three explanations of resistance in a circuit is going to be the most effective to use and how would you know when to use each one and how would you know what questions to ask to assess each?” […] That is the level that we need a lot more of*.

The gap between research and practice in education is also fostered by the differences in conceptualisation between stakeholders of what it means to be evidence-informed. By the standard of research bodies, who prioritise evidence based on robust statistical inference and experimental designs which can indicate that a certain intervention *caused* a change in identified outcomes based on a priori theory and predictions, the usual kinds of evidence-informed practice which happen in classrooms fall far short of expectations. However, it is undeniable that a teacher who is led by observation and practice-based evidence, intuitively following the needs of their students, may be an excellent teacher who supports positive outcomes well into the future. It is also undeniable that another teacher may take the same approach and either detriment their students or simply not be as effective as they could be. This is the crux of the issue, and why a theory-informed approach is generally recommended to help guide teachers and improve outcomes for all learners. Simultaneously, while never intended, it is all too easy to alienate educators from theory and research through the implication that their own work or knowledge is insufficient without external advice^68^.

To bridge this epistemological tension, concrete models such as design-based research (DBR), implementation science, and research-practice partnerships (RPPs) offer promising pathways. DBR involves iterative cycles of design, enactment, analysis, and redesign in real educational contexts, fostering collaboration between researchers and practitioners to generate usable knowledge^69^. Implementation science focuses on strategies to promote the uptake of evidence-based practices, addressing barriers like contextual fit and sustainability to close the ‘know-do’ gap^70^. RPPs operate on the principle of mutual benefit, where researchers gain insights into real-world contexts, enhancing the relevance of their research, while practitioners gain access to research expertise that can inform and improve their practice^71^. The relationship is inherently collaborative, necessitating equal engagement from both parties, which can lead to sustainable improvements in practice and the development of innovative solutions to entrenched challenges faced by practitioners^72,73^. These models align with our workshop discussions on participatory methods and could enhance methodological rigour while respecting practice-based insights.

This issue is further entrenched by lack of understanding on the part of researchers, whose theoretical knowledge may prove insufficient or impractical to meet classroom needs^74–77^, posing challenges for conducting research in applied settings which will be discussed in the later section on methodologies. The failure to properly merge theory with practice has led to a general ‘best bets’ approach^34^, which some may consider acceptable and others (particularly in the research world) totally unacceptable. This creates a tension which stifles collaboration, because it is easier for parties to stay in their own camps.

Another implication is the limitation of current research methods in terms of modelling complexity. The predominant use of RCTs over the past decade means that complex educational phenomenon are often oversimplified out of necessity to meet the requirements of the experimental model. In addition, there have been few mixed-methods or multi-strand intervention evaluations which truly examine the implementation side and take the aforementioned role of context into account (see Outhwaite et al^78^. for a proposed alternative evaluation approach and case example). In this area, a tension exists between macro and micro levels, between what researchers and educators intend to achieve in the broader context of the field, and the small-scale decisions about what can be measured and how. Situations in educational intervention research in which randomisation is not possible or the case-control design is unethical are so routine that every educational researcher is aware of them, but so far, there has been very limited discussion over how to address these issues^79^.

Our discussions highlighted differences in structural mechanisms for supporting research between countries which can impact these methodological issues and the kinds of research questions that are addressed. In the United Kingdom, there is a clear pipeline to scale interventions up for an RCT, but limited infrastructure (funding-wise and institutionally) to handle larger questions about interdisciplinary strategising to advance the field as a whole. In France, there is a highly robust centralised system for administrative data from schools, but no comparative structure for RCTs. In Chile, lack of governmental standardisation of evidence requirements for use in schools is an issue, echoed in the UK by the lack of an educational equivalent of National Institute for Health and Care Excellence (NICE) guidelines in medical practice. These guidelines provide evidence-informed, co-produced recommendations for practitioners regarding how to work with different health conditions^80^. A cyclical issue is evident here, with lack of consensus and evidence standards failing to inform policy, and policy then unable to produce coherent top-down strategies for evidence standards and integration, feeding back into lack of consensus, and so forth.

One final methodological issue which also feeds into theoretical gaps is the paucity of measurements which can track outcomes across different age ranges. The poor availability and quality of standardised measurements for a variety of educational outcomes (such as maths outcomes^81^) encourages a focus on short-term rather than long-term outcomes and prevents a full developmental picture from being taken, as well as the identification of trajectories. These limitations affect the acquisition of longitudinal datasets that may inform the impact of teaching policies and practice on student outcomes. The development of reliable measurements which are capable of facilitating a developmental focus is therefore another area which should attract the attention of research and funding if we wish to progress. Since 2019, the Ministry of Education in France has conducted nation-wide literacy and numeracy assessments of all students in Grades 1 and 2. Progressively, this has been extended to all grades to cover the entire population of students from Grade 1 to Grade 10 (starting in 2024). All testing material is adapted for SEND students. This will allow researchers to progressively study the trajectory of students’ learning across all grades including for SEND students. That said, the quality of the assessment remains largely unstudied and the assessment is again restricted to academic skills relevant for subjects such as literacy and mathematics, neglecting a broader range of school subjects and ‘soft skills’ such as critical thinking.

### Gaps in empirical testing and evidence verification

Lack of ability to replicate findings—the challenge identified as the ‘replication crisis’ (see Hillary and Medaglia^82^ for a discussion relevant to intervention studies) – is an issue which feeds poor consensus and theoretical development^79^. However, replication of findings requires greater levels of experimental control than are typically possible in educational settings, as previously discussed. In addition, such tightly-controlled settings ignore the fact that cognitive systems (and therefore the ability to learn/improve educational outcomes) are highly context sensitive, i.e. learning processes may operate differently between different settings. Findings from the CEN’s *MetaSENse* systematic review of interventions for SEND students found that most interventions that received an evaluation had only been evaluated once, in one particular school or context^36^. Therefore, it is generally unclear if intervention trial findings replicate due to lack of evidence. This raises the question: how can we build a science of accumulation without replication?

The answer is unlikely to be by creating a more homogeneous context; asking educators to adapt to the demands of the programme rather than the programme adapting to the demands of the context is unrealistic. Firstly, adapting to the context requires an *understanding* of the context, which can be achieved through dialogue with educators and other stakeholders^62^. Researchers need to develop an understanding of the variables that are relevant and can be controlled for as covariates, within the bounds of what can be measured and how within the educational setting.

Secondly, a shift in focus from the sole question of efficacy/effectiveness to understanding *mechanism* and the way that mechanism *interacts with context* could provide a means to get at universality. What is it about the intervention that works, how is this principle shaped by the environment, and how can we take this principle and apply it to other situations? Again, this is an example which requires enhanced granularity and multi-level research design, but one which would serve to enhance the ability to generate useful findings and identify common educational principles which can in turn inform theory. It may also serve to support teachers’ understanding of effective implementation (i.e. ensuring that the right conditions and elements are present in order to achieve and optimise effects) and could potentially support another priority identified by teachers: understanding how different practices and interventions can work together to produce effects^39^. By assessing interventions and practices in isolation, this kind of understanding is not possible, but by taking a perspective that includes mechanism and context, we can move towards multi-level explanations and understanding of how a particular programme fits within the bigger picture of classroom practices.

### Gaps in research with specific populations

As the aim of much research is to generalise from a small sample population to a wider population, this can sometimes create a misleading impression that an intervention will work well for most students in diverse circumstances. In fact, there are many mediating individual and demographic factors which can impact how well the intervention works in specific populations of students. For example, metacognitive interventions are rated “very high impact for very low cost based on extensive evidence” in the EEF Teaching and Learning Toolkit summary for educators (https://educationendowmentfoundation.org.uk/education-evidence/teaching-learning-toolkit/metacognition-and-self-regulation). In the review that this rating was based upon, however, evidence for metacognitive intervention effectiveness in low socioeconomic status (SES) populations appeared inconsistent and weak^83^. It is commonly suggested that low-SES demographics would benefit the most from metacognitive interventions, as they are believed to experience less opportunities to develop metacognitive skills^83^—yet the small amount of available evidence for metacognitive theory and interventions applied to this group means we do not know how these processes and interventions work in this population, and so it is not possible to say this with certainty.

In general—not just within metacognition—there are few studies which examine intervention effects and processes in low-SES groups, perhaps due to the difficulty with accessing these participants. The research world still suffers from an emphasis on findings from WEIRD (White, Educated, Industrialised, Rich, Democratic) samples, despite signs of variation in the broader population that need to be understood to build bigger models and theories. For example, there is evidence that children in South African townships have high levels of executive functioning^84^, despite the general consensus that low SES is associated with low executive functioning^85–87^. In addition, there is a stronger relationship between non-symbolic number ability and later maths achievement in West Africa compared to Western children^88^. With compounding evidence to suggest that low-SES children may develop better cognitive skills to cope with environmental demands^89,90^, the role of social disadvantage needs to be considered in a nuanced way as conveying potential advantages and disadvantages which are not evenly applied across populations, neither within nor between countries. More research testing effects, particularly those broadly known or well-accepted within WEIRD samples, in the Global South would constitute a step forward in this regard.

Another area which urgently requires greater attention is research into learning and education for lesser-studied SEND conditions, for whom what works in the classroom has largely not been evaluated^36^. For example, research is only just beginning to emerge on neurolinguistic development in children with language delay^91^ which could inform interventions with Developmental Language Disorder (DLD)^92^. This is despite the prevalence of DLD in the United Kingdom being estimated at just under 10%^93^, equivalent to the oft-quoted 10% prevalence rate in dyslexia^94^. Despite these equivalent prevalence estimates, the difference in the amount of attention these two language-based disorders have received is staggering. And again, the interactions between these conditions and demographic factors such as SES are virtually unexplored.

In addition, when interventions are studied, the study’s focus is usually on ‘what works’, not ‘what *doesn’t* work/produces worse outcomes’ nor ‘what works equally well to teaching as usual’, particularly in these niche populations. This can be due to publication bias, including the preference for publishing statistically significant rather than null findings^95^. For a full picture of what constitutes effective educational support—for all students, not just for SEND—null findings must be published and also interpreted appropriately; that is, a null finding does not indicate that the programme ‘didn’t work’, but rather that it worked equally as well as teaching as usual. There may be other reasons to take on alternative practices in these situations, such as improved cost-effectiveness.

In order to feed into the knowledge base of the science of learning, increased attention should also be given to the reasons *why* something may not have demonstrated enhanced effects over teaching as usual in a particular sample or within a particular context, especially if the intervention was backed by a robust theory of change. Further, more widespread study of potential *harms* of interventions in educational contexts must be pursued. We cannot simply assume that interventions are harmless — this must be tested. When it is tested, this may expose certain subpopulations for whom the intervention appears to produce a detriment, as was the case in the MYRIAD trial^10^, which found that a universal mindfulness intervention in schools appeared to be ineffective at promoting better well-being outcomes for the majority of students, and detrimental to mental health outcomes for participants with pre-existing higher levels of internalising symptoms. To address these issues, more attention needs to be paid in research evaluations to explicitly investigating and reporting effects in sub-populations and examining the role of contextual factors/implementation factors that can make an intervention more or less effective.

## Discussion

This gap analysis combined international perspectives from five leaders of labs involved in the science of learning in order to identify limitations in the current sphere of knowledge, research priorities, methods and infrastructure which present challenges for integrating research evidence in classroom settings. An adapted five-dimensional version of the research gap analysis framework developed by Miles^23^ was used to structure thematic analysis of presentation and discussion content. The first identified dimension concerned *gaps in existing theory and knowledge*, which highlighted a stagnation of educational theory, the continued prevalence of myths about learning, and the unevenness of how much literature exists concerning different school subjects and populations of learners. *Gaps between research and practice* elaborated on the difficulty with creating pedagogical approaches that generally support learning for all students due to the lacking knowledge base and considered the practical problems of research literacy and access to primary research and/or research summaries for educators. *Gaps in research methodologies* discussed issues with over-reliance on RCTs in educational literature and proposed some alternative methods that may better address the complexity of learning in classroom settings, whilst advocating for participatory methods to ensure that research is addressing questions and topics that are of use to educators. *Gaps in empirical testing and evidence verification* concerned issues with accumulating evidence and reaching consensus in the context of the replication crisis, which in education concerns an absence of replication studies more often than a failure to replicate. Finally, *gaps in research with specific populations* gave examples where effects observed in WEIRD populations do not appear consistent with examples from the Global South, challenging what might otherwise be taken as universal concepts about how learning happens. This final section called for more research into lesser-studied populations and for the publication of null findings – to combat publication bias and to better establish what practices are effective.

The first apparent takeaway from this gap analysis is that many of these challenges are largely shared in the educational research landscapes in different countries, as we were able to reach consensus for the relevant issues on each of the areas in the applied framework. This finding is at once sobering and bolstering, for while it appears that similar challenges exist on an international basis (and none are easy fixes), there is a clear opportunity for international collaborations to work together to achieve solutions. There are contextual nuances in different countries to which solutions may need to adapt, but by discussing our respective limitations and strengths, we can use knowledge exchange to identify productive ways forward that can potentially benefit researchers and practitioners on an international basis.

Integrating research evidence into classroom practices is a complex, multi-level issue that requires cooperation from multiple systems and the alignment of policy, research institutions and educational leadership to bring to fruition. Perhaps one of the most essential findings from this analysis concerns the importance of making effective integration of research evidence into practice the goal from the outset of research design. This means that dialogue and partnership with teachers, school leaders and other stakeholders about what their priorities are and what types of knowledge they need to make decisions needs to be at the centre of future research. A recent OECD working paper gave three examples of research-practice initiatives in Norway, the United States and Germany^96^. Each of these three cases tackled the same issue of cultivating research partnerships and evidence integration in different ways: a national scheme attempting to cross bridges of different governing systems between institutions; a professional development network for teachers; and a training scheme for early career researchers and teachers to broker conversation and knowledge exchange between the professions. These examples highlight that it is possible to tackle these issues from very different angles—but ultimately, this gap analysis suggests that coordinated efforts to reshape the approach towards evidence integration are necessary both between and within countries to make real change.

While the solutions outlined in this analysis are practical and grounded in workshop consensus, their implementation faces significant barriers that must be acknowledged for a realistic perspective. Securing funding for replication studies or research focused on lesser-studied populations, such as those with SEND, remains challenging due to biases in grant allocation favouring novel, high-impact projects over confirmatory or niche research^97,98^. For instance, an analysis of U.S. Institute of Education Sciences grants found that only a small fraction explicitly supported replication efforts, highlighting systemic underfunding that limits evidence accumulation^97^. Overcoming entrenched research and publication biases poses another hurdle, as negative or null results—crucial for a complete evidence base—are often underreported due to preferences for significant findings, inflating effect sizes in meta-analyses and distorting policy decisions^99,100^. This bias is particularly acute in special education, where publication tendencies favour positive outcomes, complicating efforts to publish null findings or studies on underrepresented groups^99^.

Finally, systemic policy constraints, such as misaligned incentives and bureaucratic hurdles, resist evidence-informed reforms by prioritising short-term accountability over long-term integration^101,102^. For example, policy frameworks often fail to support co-produced research or interdisciplinary collaborations, perpetuating gaps despite growing awareness^101^. Addressing these requires advocacy for policy shifts, such as dedicated funding streams and bias-mitigation protocols, to enhance feasibility on an international scale.

To summarise proposed solutions for each framework area: Firstly, more funding for lesser-researched areas, including learning in adulthood/learning across the lifespan is required to address *gaps in knowledge*. To address *gaps between research and practice*, more theory-informed, integrative and multi-level pedagogical approaches are required, for example through the development and implementation of child-centred models that connect curriculum themes across disciplines to foster interconnected learning^103^. Training and professional development opportunities are necessary for educators to develop research literacy skills, and for researchers to understand the challenges and priorities of classroom contexts. Collaborative partnerships between research and educational institutions represent another step forward, as well as accessible summaries of research evidence with practical implications for practice, such as the Education Endowment Foundation’s Teaching and Learning Toolkit, which offers evidence-rated guides and has supported teacher engagement through integrated professional development^103,104^. Likewise, to address *gaps in research methodologies*, research should integrate co-production using participatory methods to ensure that research is shaped around the needs of stakeholders. Mixed-methods process and impact evaluations of classroom interventions can develop a more robust conceptualisation of mechanisms, context and impact. There is also a key role for the development of quality measurement tools for educational outcomes, including ‘soft skills’ such as critical thinking and communication.

To address *gaps in empirical testing and verification*, researchers should aim to conduct multiple evaluations of educational interventions, either to test replication or to test effects across different settings and with different populations of students. Finally, to address *gaps in research with specific populations*, effects established in WEIRD populations should be tested with populations from the Global South, and more funding is needed for research with lesser-studied populations of SEND. Moreover, evaluations with null findings must be published to counter publication bias and give an accurate picture of ‘what works’ and published evaluations should explore reasons *why* an intervention may have produced the observed results—such as implementation or contextual factors.

While this study benefits from a pooling of the combined expertise of leading researchers in the field of educational neuroscience, this was a small and non-random sample of heads of research labs, which may introduce selection bias and limit generalisability. This study also did not benefit from the inclusion of educator perspectives in the data sample and analysis; a co-produced version of this gap analysis may be an important next step for future research.

In terms of the efforts to capture international perspectives, it must be noted that three of the five heads were from the same country and there was a lack of representation from areas such as North America. While the current study evidenced that gaps were similar between countries despite differences in context, including representative voices from more countries in the future may allow for more diverse perspectives and potentially greater room for identification of differences and similarities in best practices and proposed solutions.

In summary, this paper presented an analysis of gaps contributing to poor integration of research in classroom practices. Five heads of labs involved in the science of learning in the United Kingdom, France, and Chile met to present their work and discuss the factors contributing towards poor integration. Thematic analysis of slides and meeting notes produced themes which were organised within an adapted five-dimensional version of the research gaps framework provided by Miles^23^. This analysis produced insights on gaps and possible solutions in a range of areas, from improving the theoretical base for education and addressing research-practice gaps, to improving research methodologies and accumulating evidence. International perspectives highlighted points of learning from shared and dissimilar experiences across different country contexts. Overall, there appears to be a need for coordinated action on the part of researchers, research funding bodies, practitioners, and policymakers alike in order to bring the worlds of educational research and practice into alignment.

## Methods

Five heads of research labs engaged in the science of learning and three junior researchers from the United Kingdom, France, and Chile came together at [redacted for anonymisation purposes] to discuss current gaps for integrating research evidence into the classroom and ways to meet them. Discussions took place as a two-day follow-up workshop to an international roundtable event on challenges and solutions for classroom interventions (see Bowen et al.^105^ for the main conference report). The labs involved are well established within the field, and the heads have lengthy experience of critical analysis of the priorities for and organisation of research and translation efforts within it. All three junior researchers were involved in note-taking; two had no further involvement, and one took on the role of primary author for this gap analysis.

To begin, the five heads presented the current work, priorities and challenges for their labs in terms of developing and integrating evidence into education. These presentations and notes on the resulting discussions formed the dataset for the gap analysis. Audio recordings of presentations and conversations were not used, only textual data from slides and notes. The seven-dimension deductive research gap analysis framework proposed by Miles^23^ was then used as an organising structure, alongside the reflexive thematic analysis method outlined by Braun and Clarke^106^, to identify specific gaps where more work is needed to integrate research into the classroom. Areas where crossovers in priorities existed and common challenges were identified through thematic analysis of notes and presentation slides. Themes were identified based on frequency, cogency, and relevance of data. Areas of tension or divergence were also captured and incorporated within the thematic representations.

**Fig. 1:**
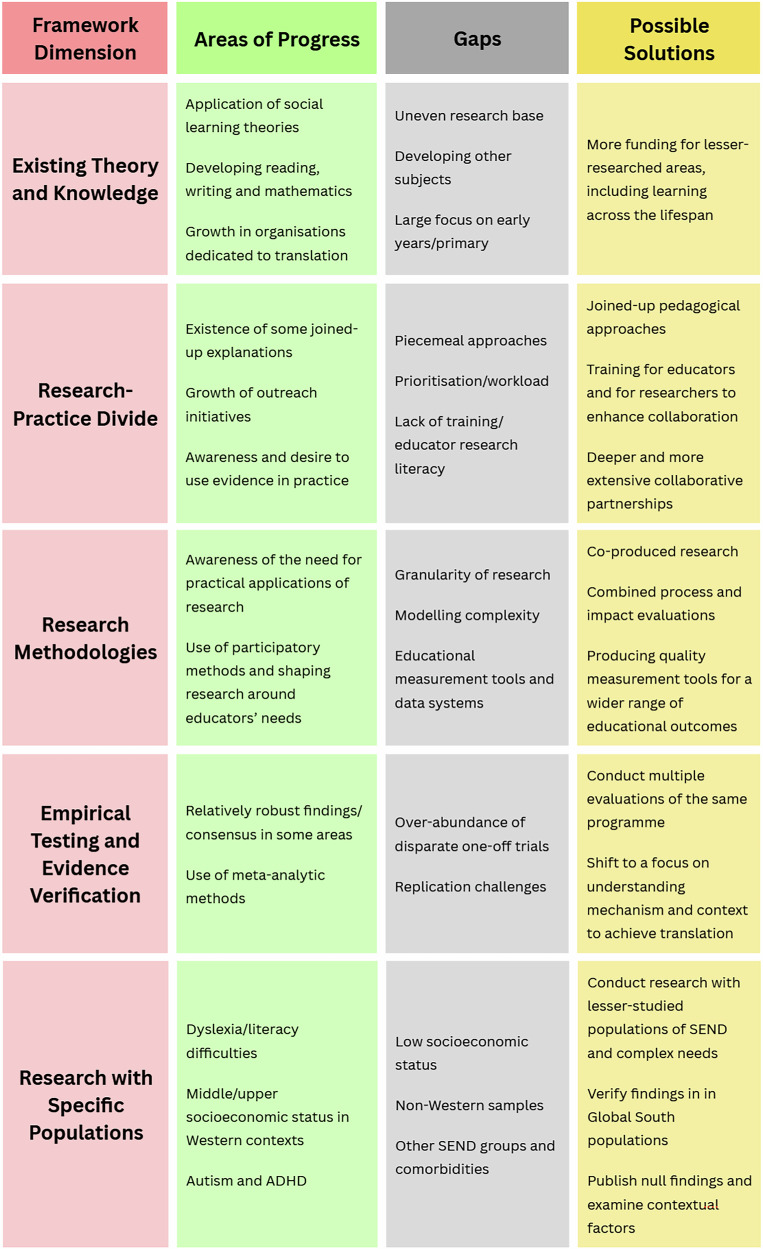
Summary of current areas of progress, gaps, and possible solutions for each gap analysis framework dimension. This figure presents a summary of key points for each of the five framework dimensions, indicating areas of progress, current gaps, and possible solutions identified during analysis.

After initial coding of the presentation notes and slides as well as notes made during discussions, the organising framework was adapted to streamline the categorisation of issues (Table 1): “Knowledge” and “Theoretical” gaps were collapsed into a single dimension, and “Empirical” and “Evidence” gaps were collapsed into a single dimension, both due to overlapping content and themes across these topic areas. In the end, gaps were organised into a five-dimension framework: (1) gaps in existing theory and knowledge; (2) gaps between research and practice; (3) gaps in research methodologies; (4) gaps in empirical testing and evidence verification; and (5) gaps in research with specific populations. This process began with all authors jointly during the live workshop and was completed afterwards by the primary author and via follow-up email exchanges, with final themes agreed between all authors Fig. 1

**Table 1:** Outline of the original Miles (2017) seven-dimension framework alongside the adapted five-dimension framework for the current study

Seven-dimension framework	Five-dimension framework
**Knowledge gap**	**Gaps in Existing Theory and Knowledge**
Definition: *Desired research findings do not exist*.	
**Theoretical gap**	
Definition: *Theory should be applied to certain research issues to generate new insights. There is lack of theory thus a gap exists*.	
**Practical-knowledge gap**	**Gaps in Research and Practice**
Definition: *Professional behaviour or practices deviate from research findings or are not covered by research*.	
**Methodological gap**	**Gaps in Research Methodologies**
Definition: *A variation of research methods is necessary to generate new insights or to avoid distorted findings*.	
**Empirical gap**	**Gaps in Empirical Testing and Evidence Verification**
Definition: *Research findings or propositions need to be evaluated or empirically verified*.	
**Evidence gap**	
Definition: *Results from studies allow for conclusions in their own right, but are contradictory when examined from a more abstract point of view*.	
**Population gap**	**Gaps in Research with Specific Populations**
Definition: *Research regarding the population that is not adequately represented or under-researched in the evidence base or prior research*.	

This table presents the original seven-dimension framework with definitions for each dimension alongside the adapted five-dimension framework illustrating how the seven dimensions were collapsed to form the new dimensions.

Due to the nature of this study wherein the data was both generated and analysed by experts in research, the views captured within the dataset are based upon the sum of a wealth of existing research and knowledge. All claims presented within the results were present within the dataset, but relevant associated literature has also been provided within the results section to establish the foundations of claims and support the robustness of findings.

## Data Availability

Data is not publicly available but can be made available upon request to the primary author (a.bowen@bbk.ac.uk).
